# Insights into the Mechanism of Tryptophan Fluorescence Quenching due to Synthetic Crowding Agents: A Combined Experimental and Computational Study

**DOI:** 10.1021/acsomega.3c06006

**Published:** 2023-11-13

**Authors:** Carl J. Fossum, Benjamin O. V. Johnson, Spencer T. Golde, Alexis J. Kielman, Brianna Finke, Macey A. Smith, Harrison R. Lowater, Bethany F. Laatsch, Sudeep Bhattacharyya, Sanchita Hati

**Affiliations:** Department of Chemistry and Biochemistry, University of Wisconsin-Eau Claire, Eau Claire, Wisconsin 54701, United States

## Abstract

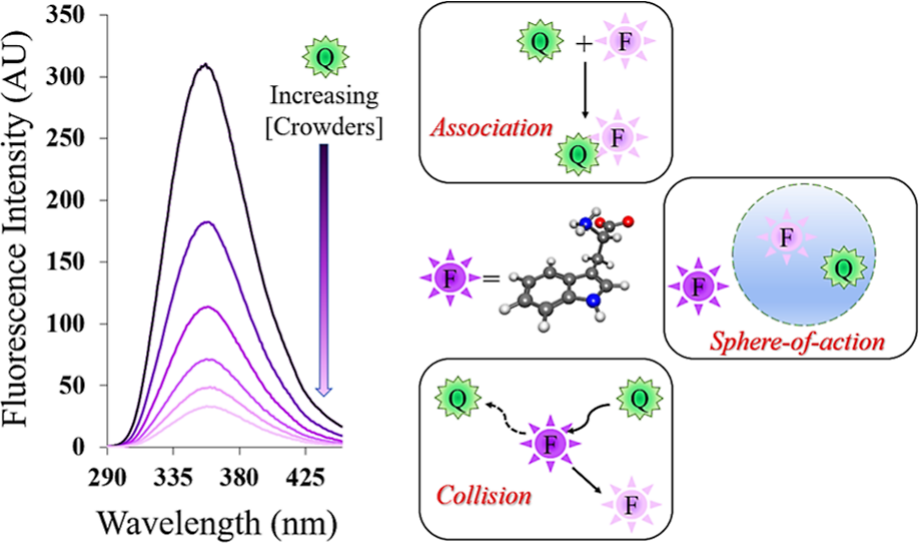

Intrinsic tryptophan fluorescence spectroscopy is an important tool for examining the effects of molecular crowding and confinement on the structure, dynamics, and function of proteins. Synthetic crowders such as dextran, ficoll, polyethylene glycols, polyvinylpyrrolidone, and their respective monomers are used to mimic crowded intracellular environments. Interactions of these synthetic crowders with tryptophan and the subsequent impact on its fluorescence properties are therefore critically important for understanding the possible interference created by these crowders. In the present study, the effects of polymer and monomer crowders on tryptophan fluorescence were assessed by using experimental and computational approaches. The results of this study demonstrated that both polymer and monomer crowders have an impact on the tryptophan fluorescence intensity; however, the molecular mechanisms of quenching were different. Using Stern–Volmer plots and a temperature variation study, a physical basis for the quenching mechanism of commonly used synthetic crowders was established. The quenching of free tryptophan was found to involve static, dynamic, and sphere-of-action mechanisms. In parallel, computational studies employing Kohn–Sham density functional theory provided a deeper insight into the effects of intermolecular interactions and solvation, resulting in differing quenching modes for these crowders. Taken together, the study offers new physical insights into the quenching mechanisms of some commonly used monomer and polymer synthetic crowders.

## Introduction

Fluorescence spectroscopy is a powerful technique for biochemical analysis.^1^ It is a very sensitive, fast, and inexpensive method for accurately determining the concentration of an analyte in a solution. By following changes in fluorescence properties, protein–ligand interactions can be studied qualitatively as well as quantitatively in equilibrium conditions.^2−9^ Moreover, because of its sensitivity to changes in local environments, fluorescence spectroscopy is used to study protein dynamics and conformational changes occurring due to alterations in the surrounding environment.^10,11^ Specifically, intrinsic fluorescence spectroscopy using the highly sensitive Trp residues has become a popular tool for obtaining static and dynamic information on a protein structure from any external perturbations as it does not require fluorescent labeling.

The interior of a cell is extremely crowded,^12,13^ and intrinsic Trp fluorescence has become a vital tool to gain a molecular-level understanding of molecular crowding effects on the structure and function of proteins because of its robustness, high sensitivity, and noninvasiveness.^14−19^ Many of these intrinsic Trp fluorescence studies involve synthetic crowders of varying chemical properties, sizes, and shapes to create a specific crowding environment, whose impact on a certain biological function is being probed. In particular, to mimic the crowding effects of surrounding biomolecules on an enzyme function, *in vitro* studies are usually performed using polymer crowding agents such as polyethylene glycols (PEGs), polyvinylpyrrolidone (PVP), dextran, or ficoll. As a part of our continued investigation on the functional domain dynamics^20 −24^ and its interplay with molecular crowding,^18^ the intrinsic fluorescence spectroscopy is being used to probe prolyl-tRNA synthetases—a structurally diverse class of multidomain proteins involved in protein biosynthesis in all three kingdoms of life.^25,26^ As revealed in our earlier studies, synthetic crowders may induce conformational changes, which in turn can increase solvent accessibility of tryptophans.^18^ Other groups have also reported intrinsic fluorescence of Trp as a probe to observe the impact of synthetic crowders on refolding and aggregation of the protein.^27,28^ The solvation effect is known to be central in shaping the crowders-induced quenching.^29,30^ Therefore, a quenching study of free Trp due to crowders is crucial to discern the effect of solvents from the effect of an aqueous protein environment. However, little is known about the impact of commonly used synthetic crowders on the fluorescence properties of the free tryptophan itself.

Herein, a study of the effects of synthetic crowding agents, both monomers and their polymers, on the intrinsic fluorescence of free Trp in an aqueous solution is presented. The condition mimics a Trp residue, which is close to the protein surface and more accessible to the solvent compared with a Trp residue in a folded protein. Both, fluorescence intensity and emission wavelength were analyzed with the expectation that the extent of quenching or enhancement of the intrinsic fluorescence may shed light on the nature of interactions between crowders and free Trp in an aqueous solution. Additionally, interactions between the indole ring of Trp and the monomer and polymer crowders were examined at the electronic level by computing the electronic structure of these complexes.

## Materials and Methods

All reagents [dextrose, dextran 40 (molecular weight: 40,000 g/mol), sucrose, ficoll 70 (molecular weight: 70,000 g/mol), ethylene glycol (EG), 1-ethyl-2-pyrrolidone (EP), and PVP 40 (molecular weight: 40,000 g/mol)] were purchased from Sigma-Aldrich, except for PEG 8 (molecular weight: 8000 g/mol), which was obtained from Fisher Scientific.

### Fluorescent Spectroscopy

Fluorescence measurements were conducted with a Cary Eclipse Fluorescence Spectrophotometer (Agilent Technologies, USA) at room temperature by exciting the samples at 280 nm (λ_max_) and recording the emission spectra from 290 to 450 nm. The spectral slits were 5 nm for both excitation and emission. All measurements of sample solutions were corrected using their respective blank solutions. In particular, the blank solutions contained all reagents but Trp (i.e., crowding agent, phosphate buffer, and sodium chloride). The samples were prepared using 1 mM stock solution of Trp in 30 mM phosphate buffer (pH = 7.4) and 100 mM NaCl to yield a final Trp concentration of 10 μM. The concentration of crowding agents was 300 mg/mL for all but PVP 40; PVP 40 concentration was maintained at 100 mg/mL because of significant fluorescence quenching. Crowding agents used in the present study are dextrose and its polymer dextran 40, sucrose and its polymer ficoll 70, EG and its polymer PEG 8, and EP and its polymer PVP 40. Before fluorescence measurements, the solutions were equilibrated at room temperature for 30 min. All measurements were performed in triplicate using a quartz cuvette with a 1 cm optical path length. The changes in fluorescence intensity and wavelength in the presence of crowding agents were monitored. The barycentric mean fluorescence wavelength (λ_bcm_) was calculated using the following eq (eq 1)
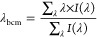
1

In the above equation, λ is the wavelength and *I*(λ) is the emission intensity at a given wavelength. The change in λ_bcm_ [Δλ_bcm_ = λ_bcm_(with crowders) – λ_bcm_(without crowders)] due to the presence of crowders was examined to monitor changes in the Trp’s local environment.

### Concentration and Temperature Variation Studies

Most traditional fluorophores encounter fluorescence quenching in the presence of crowder molecules. Quenching (decrease in fluorescence intensity) can occur due to collisions during the excited state lifetime (“hard interaction” or dynamic quenching) or it may occur due to the formation of complexes through noncovalent interactions between fluorophores and quenchers in the ground state (“soft interactions” or static quenching).^31,32^ To examine if the Trp fluorescence quenching process is static, dynamic, or both in the presence of crowders, a series of aqueous solutions with 10 μM Trp and varying crowder concentrations (50–300 mg/mL; concentration increased by 50 mg/mL) in 30 mM phosphate buffer (pH = 7.4) and 100 mM NaCl were prepared and equilibrated at room temperature for 30 min. All fluorescence measurements were performed in triplicate at room temperature. The quenching data were analyzed using the Stern–Volmer model^4^
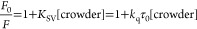
2

In eq 2, *F*_0_ and *F* are the fluorescence intensities observed in the absence and presence of crowder, respectively, *K*_SV_ is the Stern–Volmer quenching constant, *k*_q_ is the bimolecular quenching rate constant limited by the rate of diffusion of molecules, and τ_0_ is the excited state lifetime in the absence of quencher. For dynamic quenching, the Stern–Volmer constant in eq 2 can be replaced by *K*_D_, the dynamic quenching constant
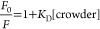
3

Similarly, the Stern–Volmer equation for static quenching is expressed as
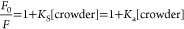
4

*K*_S_ is the static quenching constant of the complex, which as discussed by Genovese et al.^33^ can be equated to the association constant, *K*_a_, under the assumption of negligible concentration of fluorophore compared to that of quenchers (crowders). If either dynamic or static quenching occurs, a plot of  versus [Crowder] should yield a linear plot with a slope equal to *K*_SV_, which could be equated to *K*_D_ or *K*_S_. If both static and dynamic quenching occur simultaneously, a nonlinear Stern–Volmer plot is expected to be observed as expressed by the following equation
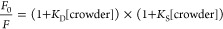
5

Equation 5 can be expressed in the following quadratic equation
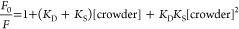
6

The above eq 6 can provide the dynamic (*K*_D_) and static (*K*_S_) quenching constants. Temperature effects can be used to distinguish between the two forms of quenching. The dynamic quenching will increase with temperature as the diffusion rates of the quencher and fluorophore tend to be faster at a higher temperature. In contrast, static quenching tends to be lower at higher temperatures as complex formation strength is inversely proportional to temperature. The nonlinear Stern–Volmer plot could also be due to the sphere-of-action mechanism, where the quencher and fluorophore may not necessarily form a complex but are in close proximity within a spherical region so that fluorescence is quenched immediately upon excitation.^34^ The sphere-of-action mechanism is expressed by eq 7
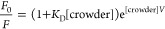
7

In eq 7, *V* is the volume of the sphere surrounding the fluorophore within which the probability of quenching is unity.

To identify the quenching mechanism, we performed fluorescence experiments at different temperatures. First, the temperature variation experiments were conducted using 10 μM Trp and 300 mg/mL crowders by varying the temperature from 25 to 70 °C in 5 °C increments. Then, the concentration variation study was performed at 25 and 50 °C, and Stern–Volmer constants (*K*_SV_) were determined. Microsoft Excel (Version 2304) and R (Version 4.1.3) were used for carrying out linear and nonlinear regressions, respectively. The coefficient of determination (*R*^2^), computed from the square of the correlation coefficient, was used as a goodness-of-fit measure for fitted models.

### Gibbs’ Free Energy of Binding Calculations Using Density Functional Theory

To obtain the molecular-level understanding of the type of interactions observed between the Trp fluorophore and the four synthetic monomer crowders and their respective polymers, the solvated Gibbs’ free energy of binding of each crowder–fluorophore complex was computed. For monomer crowders, beginning with a neutral indole ring, a single crowder molecule was placed above the conjugated ring using Avogadro.^35,36^ Following the system setup, geometry optimizations and frequency calculations were performed using Gaussian 16.^37^ The optimized structure and Gibbs’ free energy of binding for each synthetic crowder complex were calculated at the level of Kohn–Sham density functional theory (DFT).^38^ The density functional M06-2X^39,40^ was chosen for its reliable ability to model medium-range noncovalent interactions. A smaller basis set 6-31+G(d,p)^41^ was used in all calculations. Solvation effects were modeled using implicit Solvation Model based on Density (SMD).^42^ The highest occupied molecular orbitals (HOMOs) were generated using IQmol Molecular Viewer.^43,44^

Polymer crowders were also built using Avogadro.^35,36^ A single Trp molecule was inserted into the cavity generated by polymer crowders, and the geometry of these complexes was optimized using molecular mechanics and the Universal Force Field,^45^ available in the Avogadro program. These complexes were then subjected to further optimization followed by frequency calculation using the same set of functionals as used for monomer crowders, namely, M06-2X.

**Scheme 1:**
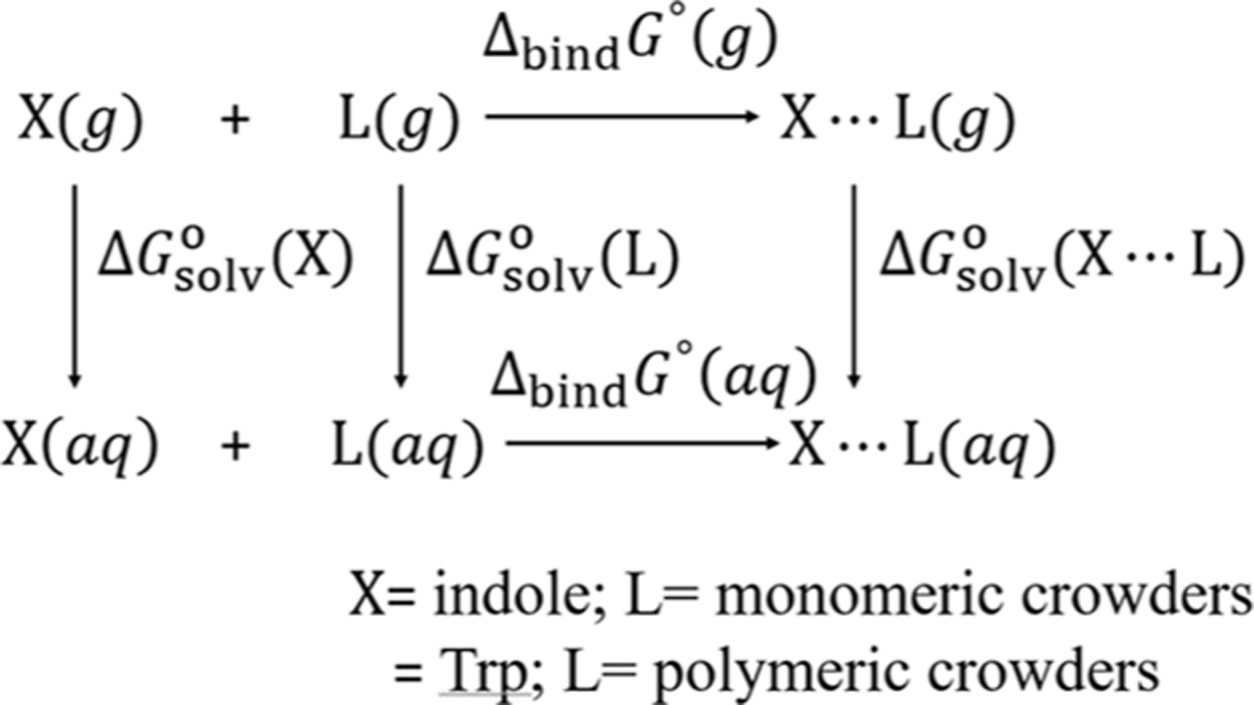
Thermodynamic Scheme for Computing the Gibbs Free Energy of Association between Crowders and Tryptophan

As illustrated in Scheme 1, gas-phase Gibbs’ binding free energy, Δ_bind_*G*° (g), for indole-monomeric crowder and Trp-polymeric crowder complexes were calculated using eq 8
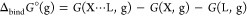
8

Here, *G*(X···L, g) represents the gas-phase Gibbs’ free energy of the indole-monomeric crowder or Trp-polymeric crowder complexes. Likewise, *G*(X, g) and *G*(L, g) represent the individual free-energy contributions of indole (or Trp) and any given synthetic crowder, respectively.

Following Scheme 1, the aqueous-phase Gibbs’ free energy of binding between indole (or Trp) and the respective synthetic crowder, Δ_bind_*G*° (aq) (Scheme 1) was then calculated using eq 9
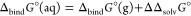
9where ΔΔ_solv_*G*° quantity is equal to the difference of the solvation free energies of the complex and the free species in Scheme 1 (eq 10)
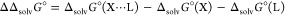
10

## Results and Discussion

Of the three aromatic amino acids, emission from Trp is particularly useful for intrinsic fluorescence spectroscopy because of its large absorptivity, quantum yield, and overall high sensitivity to the local environment.^4^ The indole ring of Trp contains unique spectral features that distinguish it from other aromatic amino acids. Trp emission occurs from two electronic absorption transitions, ^1^*L*_*a*_ and ^1^*L*_*b*_.^4^ Trp emission originates from the ^1^*L*_*a*_ state in polar environments, whereas emission from the ^1^*L*_*b*_ state is predominant in completely nonpolar environments. Moreover, the ^1^*L*_*a*_ state of Trp has a large excited-state dipole moment and is more sensitive toward hydrogen bonding compared to the excited ^1^*L*_*b*_ state.

Tryptophan fluorescence parameters, namely, intensity and wavelength (λ_max_) are sensitive to its local environment.^1,4^ In particular, Trp fluorescence maximum (λ_max_) and intensity are strongly affected by the polarity of its microenvironment.^1,4^ Fluorescence quenching has been a valuable tool in gauging the location of tryptophan fluorophores within the tertiary and quaternary structures of a protein. When the indole nitrogen is exposed to a polar surrounding, a red-shift in barycentric mean fluorescence wavelength (λ_bcm_) is observed.^1^ On the other hand, a blue-shift is observed when Trp residues are excluded from polar solvents, such as when the residue is moved toward the protein interior. Fluorescence properties, namely, λ_max_ and fluorescence intensity are used to probe conformational changes in a protein due to the changes in the external environment. In the present study, the impact of monomer and polymer crowding agents on the free Trp fluorescence properties were analyzed.

### Decrease in Fluorescence Emission Intensity

**Figure 1:**
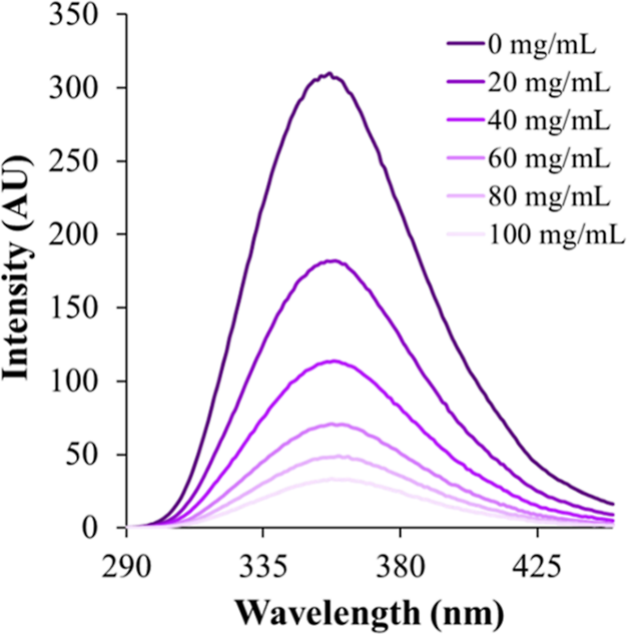
Fluorescence spectra of tryptophan (10 μM) in the presence of variable amounts of PVP 40 at room temperature. AU: arbitrary unit.

The quenching of the fluorescence intensity varied in the presence of crowders. Both crowder type and concentration have impacts on the emission intensity (Figure 1 and Table 1).

**Table 1:** Effects of Different Crowding Agents on Tryptophan (10 μM) Fluorescence Properties^a^

crowding agents	concentration (M)	*R*_h_ (Å)	percent decrease in emission intensity	Δλ_bcm_
dextrose	1.67	4.2^b^	5.7 ± 0.5	not significant^d^
dextran 40	0.00750	4.78^b^	63 ± 4	not significant^d^
sucrose	0.876	5.2^b^	27 ± 2	1.47 ± 0.07
ficoll 70	0.00429	40^b^	74 ± 0.2	not significant^d^
ethylene glycol	4.83		–19.1 ± 0.5^c^	–1.1 ± 0.2
PEG 8	0.0375	27.5^b^	not detectable	not significant^d^
1-ethyl-2-pyrrolidone	2.65		19 ± 3	–4.4 ± 0.4
PVP 40	0.0025	5.10^b^	89 ± 2	not significant^d^

a The final concentration of all crowders but PVP 40 was maintained at 300 mg/mL; for PVP 40, it was 100 mg/mL.

b Data for hydrodynamic radii were taken from the literature for dextrose and sucrose,^46^ dextran 40 and PVP 40,^47^ ficoll 70,^19^ and PEG 8.^48^

c Enhancement of fluorescent intensity was observed.

d A quantity of <1 nm was reported as “not significant” for Δλ_bcm_.

The maximum quenching was observed in the presence of 100 mg/mL PVP 40 (89%) followed by that in the presence of 300 mg/mL ficoll 70 (74%) (Table 1). Only an ∼6% reduction in fluorescence intensity was observed in the presence of dextrose. PEG 8 had the least effect on fluorescence intensity; it was barely above background noise (Table 1). No trend was observed between the hydrodynamic radius of the crowding agent and the percent reduction in the fluorescence intensity, which could be because the concentration of the crowders used was greater than their overlap concentrations.^49^ Compared to their monomers, polymer cosolutes are more effective quenchers of Trp fluorescence–dextrose (∼6% reduction) versus dextran 40 (63% reduction); sucrose (27% reduction) versus ficoll 70 (74% reduction), and 300 mg/mL EP (19% reduction) versus 100 mg/mL PVP 40 (89% reduction). The enormous reduction in fluorescence intensity of Trp in the presence of PVP 40 (Figure 1) could be due to the fact that the absorption wavelength of PVP 40 ranges from 230 to 400 nm. The PVP 40 exhibits two excitation peaks due to the presence of C=O and N≡C groups at 285 and 330 nm, respectively.^50^ Therefore, energy transfer may be taking place from the excited Trp to PVP 40, causing a significant decrease in the emission intensity of Trp. Other mechanisms such as sphere-of-action quenching (vide infra)^34^ and self-aggregation of Trp molecules in the presence of PVP 40 could also cause such a drastic reduction in fluorescence intensity.

### Shifts in Barycentric Mean Fluorescence Wavelength

As mentioned earlier, if a Trp molecule becomes more exposed to the surrounding hydrophilic solvent, there is a shift in λ_bcm_ toward a higher wavelength (red-shift).^1^ Conversely, if a tryptophan becomes less exposed to the polar solvent, λ_bcm_ undergoes a spectral shift toward a lower wavelength (blue-shift). The observed changes in λ_bcm_ (Table 1) indicate that the polymer crowders used in the present study do not induce any significant changes in the microenvironment surrounding the Trp fluorophore. For monomeric crowders, a small change in λ_bcm_ was observed for all but dextrose. A red-shift was observed for sucrose (Δλ_bcm_ = ∼1.5 nm) (Table 1), whereas a blue-shift in the fluorescence maximum was observed for monomer cosolutes EG (Δλ_bcm_ = −1.1 nm) and EP (Δλ_bcm_ = −4.4 nm), i.e., the tryptophan becomes less exposed to the polar solvent in the presence of EG and EP.

### Stern–Volmer Plots and the Quenching Mechanisms

**Figure 2:**
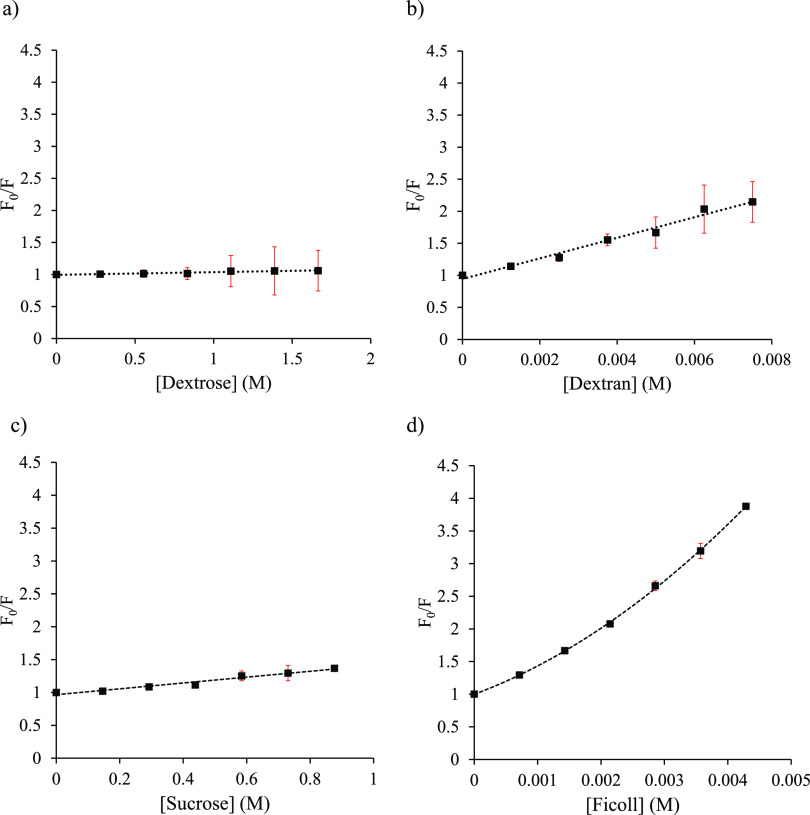
Stern–Volmer plots of tryptophan (10 μM) with varying molar concentrations of crowders at 25 °C: (a) dextrose, (b) dextran 40, (c) sucrose, and (d) ficoll 70. Dotted lines represent the line of best-fitting, and error bars are shown in red.

**Figure 3:**
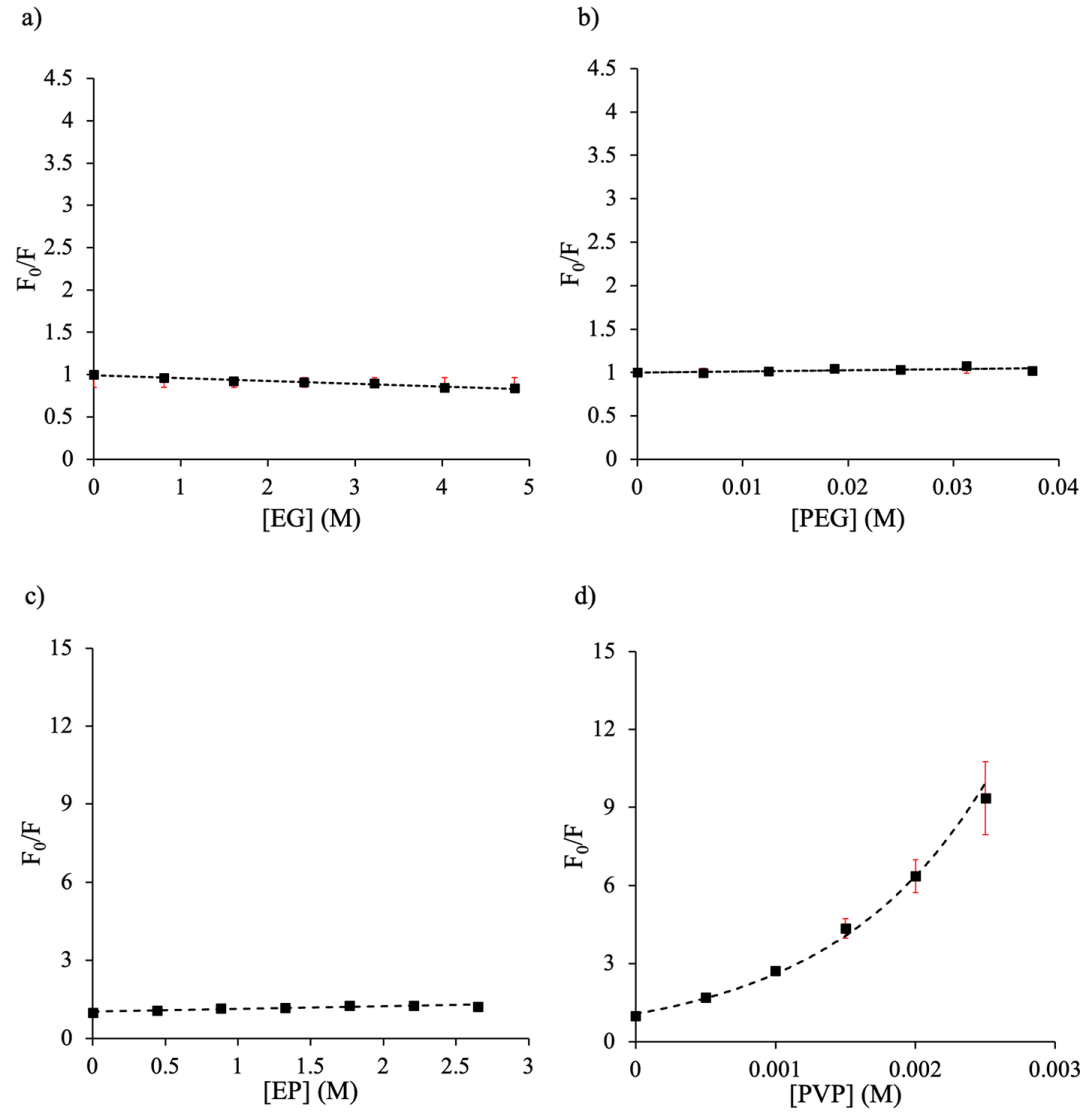
Stern–Volmer plots of tryptophan (10 μM) with varying molar concentrations of crowders at 25 °C: (a) ethylene glycol (EG), (b) polyethylene glycol 8 (PEG), (c) 1-ethyl-2-pyrrolidone (EP), and (d) polyvinylpyrrolidone 40 (PVP). Dotted lines represent the line of best-fitting and error bars are shown in red.

The quenching in fluorescence intensity of Trp fluorophores in the presence of crowders could be either due to “hard interactions” (collisional, dynamic quenching) or due to noncovalent interactions (“soft interactions”, static quenching) between crowders/solvent molecules and Trp fluorophores as well as among Trp molecules.^4^ The other possibility is the sphere-of-action quenching mechanism. To investigate the exact mechanism of quenching, Stern–Volmer plots were made. Analysis of the Stern–Volmer plots showed that the Trp fluorescence intensity decreased to varying degrees in the presence of different crowders. Figures 2 and 3 depict the quenching profiles for monomer and polymer crowders. The Stern–Volmer plots in Figures 2a–d and 3a–d (crowder concentrations are expressed in M) revealed that polymer crowders are more effective than monomers in quenching Trp fluorescence; PEG 8 is an exception (Figure 3b).

For the monomer crowders, linear Stern–Volmer plots were obtained. Among the four monomer crowders studied, sucrose is the most effective quencher with *K*_SV_ of 0.444 M^–1^ (eq 2, Table 2). Interestingly, unlike other crowders, a slight enhancement of fluorescence intensity occurs in the presence of EG (*K*_SV_ = −0.0325 M^–1^) (Table 2). For the polymer crowders, both linear and nonlinear Stern–Volmer plots were obtained (Figures 2 and 3). The linear Stern–Volmer plots (Figures 2b and 3b) suggest that the polymer dextran 40 (*K*_SV_ = 1.60 × 10^2^ M^–1^) induces either static or dynamic quenching, and PEG 8 induces a minimal quenching effect (*K*_SV_ = 1.40 M^–1^). It has been suggested that the static quenching may also produce a linear Stern–Volmer plot in some cases.^1,51^ On the other hand, the nonlinear with upward curvature plots (Figures 2d and 3d) indicate complex (static, dynamic, and sphere-of-action) quenching mechanisms for ficoll 70 and PVP 40. However, the nonlinear curve fitting using eq 6 did not produce real solutions for static and dynamic quenching constants for these two polymers. On the other hand, eq 7 produced solutions of the nonlinear regressions (Table 2) and revealed that the quenching by ficoll 70 and PVP 40 takes place via the sphere-of-action mechanism.^34^ The *K*_D_ and *V* values of ficoll 70 are 266 and 137 M^–1^, respectively, whereas the *K*_D_ and *V* values for PVP 40 are 710 and 486 M^–1^, respectively. These quenching constants (Table 2) suggested that PVP 40 is a better quencher than ficoll 70. In addition to the sphere-of-action mechanism, it is possible that Trp molecules cluster together in the presence of these polymer crowders (PVP 40 and ficoll 70). It has been reported that synthetic polymers can change the solvent properties of water when dissolved in it, which could have an impact on the aggregation of Trp molecules. The crowder-induced changes in solvent properties of water are reported to be an important factor in molecular crowding effects.^29,30^

**Table 2:** Stern–Volmer Constants for Tryptophan in the Presence of Crowding Agents^a^

	*K*_SV_ (M^–1^)		
crowders	*K*_D_ (M^–1^)	*K*_S_ (M^–1^)	*V* (M^–1^)
dextrose^b^	0.0415 (0.90)		
dextran 40^c^		160 (0.98)	
sucrose^b^	0.444 (0.96)		
ficoll 70^d^	266 (0.99)		137 (0.99)
ethylene glycol^b^	–0.0325 (0.97)		
polyethylene glycol (PEG 8)^c^	1.40 (0.42)		
1-ethyl-2-pyrrolidone^b^	0.254 (0.92)		
PVP 40^d^	710 (0.99)		486 (0.99)

a The goodness-of-fit (correlation coefficient) of the fitted functions are given in parentheses.

b *K*_D_ was obtained using eq 3.

c *K*_S_ was obtained using eq 4.

d *K*_D_ and *V* were obtained using eq 7.

These quenching results suggested that the mode of interactions between Trp and the surrounding solvent molecules and crowders varies between the monomer and polymer crowders. In most cases, monomer crowders (except for ethylene glycol) and the polymer crowder dextran 40 induce quenching via either the dynamic or static mechanism (linear Stern–Volmer plots). To further investigate the mechanism of quenching for monomer crowders and polymeric dextran 40, a temperature variation study was carried out using 10 μM Trp and 300 mg/mL crowders by varying the temperature from 25 to 70 °C in 5 °C increments; the samples were excited at 280 nm, and the maximum emission intensity between 290 and 450 nm were recorded for each temperature. If the quenching mechanism is predominantly collisional (dynamic), then fluorescence quenching would be higher at elevated temperatures as the diffusion rates of fluorophores and quenchers (crowders and solvent molecules) would increase. On the other hand, if the quenching mechanism is purely static, quenching will be less at higher temperatures; the soft interactions between Trp and crowders would be disrupted at higher temperatures, which would allow a higher fraction of Trp molecules to be excited. Figure S1 shows that as the temperature increased from 25 to 70 °C, Trp fluorescence intensity decreased even in the absence of any crowders (red line), indicating more frequent collisions between fluorophores and solvent molecules as temperature increases. In the presence of 300 mg/mL crowders, there was a gradual decrease in the fluorescence intensity with increasing temperature; quenching was higher at 70 °C compared to that at 25 °C for all crowders (Figure S1). An enhancement in Trp fluorescence in the presence of EG was evident (cyan line); the measured intensity was consistently higher than that of the buffer-only solution (red line). Although the exact mechanism for enhancement of fluorescence intensity is not well-understood, it appeared that unlike other crowders, EG sequesters the Trp fluorophores from being quenched by the polar solvent molecules.

A fluorescence lifetime measurement at variable quencher concentrations can provide information about dynamic versus static quenching,^4^ which we could not do because of the limitation of our present equipment. An alternate procedure, reported by Arık et al.^51^ demonstrates that a decrease in bimolecular quenching rate constant, *k*_q_, at elevated temperature indicates static quenching. To have a better insight into the quenching mechanism, a concentration variation study was performed at 25 and 50 °C, and Stern–Volmer plots were analyzed. Using the literature value of Trp’s mean excited-state lifetime in the absence of quencher *s* (τ_0_) of 3.1 ns,^4,52^ the biomolecular quenching rate constant, *k*_q_, values were calculated using eq 2 (Table 3). Additionally, it was assumed that τ_0_ of Trp will be half when the temperature was raised by two-folds, and therefore, τ_0_ of 3.1 and 1.55 ns were used at 25 and 50 °C, respectively, for computing *k*_q_ values.^53^ The calculated *k*_q_ value at 25 °C is the highest for sucrose among the four monomers, and it is the most efficient quencher among them. In the presence of variable amounts of monomer crowders, linear quenching profiles were observed at both temperatures, 25 and 50 °C. There was a greater than 50% decrease in Trp fluorescence intensity at 50 °C compared to that at 25 °C even in the absence of any crowders (Figure S2), which suggested that the quenching of Trp fluorescence intensity is predominantly via a collisional mechanism. The increased diffusion rates of solvent molecules and Trp fluorophores at higher temperatures may be the main factor for the quenching. The effect of increased concentration of crowders on quenching was modest (Figure S2). The Stern–Volmer plots for fluorescence quenching by monomer crowders at 25 and 50 °C are shown in Figure S3. The biomolecular quenching constant *k*_q_ values at 50 °C were computed for monomer crowders and compared with that for 25 °C. A slight increase in *k*_q_ at the elevated temperature for sucrose and EP (Table 3) indicates dynamic quenching of the Trp fluorescence intensity in the presence of these two monomeric crowders. For the monomeric dextrose, the slope of the Stern–Volmer was negative at 50 °C, and there was an enhancement in fluorescence both at 25 and 50 °C in the presence of EG.

**Table 3:** Stern–Volmer Constants and Bimolecular Quenching Rate Constant, *k*_q_, at 25 and 50 °C for Monomer Crowders^a^

	*K*_SV_ (M^–1^)		*k*_q_ (M^–1^ ns^–1^)	
crowders	25°C	50°C	25°C	50°C
dextrose	0.0415 (0.90)	–0.038 (0.74)	0.0134	
sucrose	0.444 (0.96)	0.241 (1.0)	0.143	0.155
ethylene glycol	–0.0325 (0.97)	–0.040 (0.94)		
1-ethyl-2-pyrrolidone	0.254 (0.92)	0.138 (0.84)	0.0819	0.0888

a The bimolecular quenching rate constant, *k*_q_, values were obtained using eq 2 with τ_0_ value of 3.1 ns^4^ and 1.55 ns at 25 and at 50 °C, respectively. The goodness-of-fit (correlation coefficient) of the fitted functions are given in parentheses.

**Figure 4:**
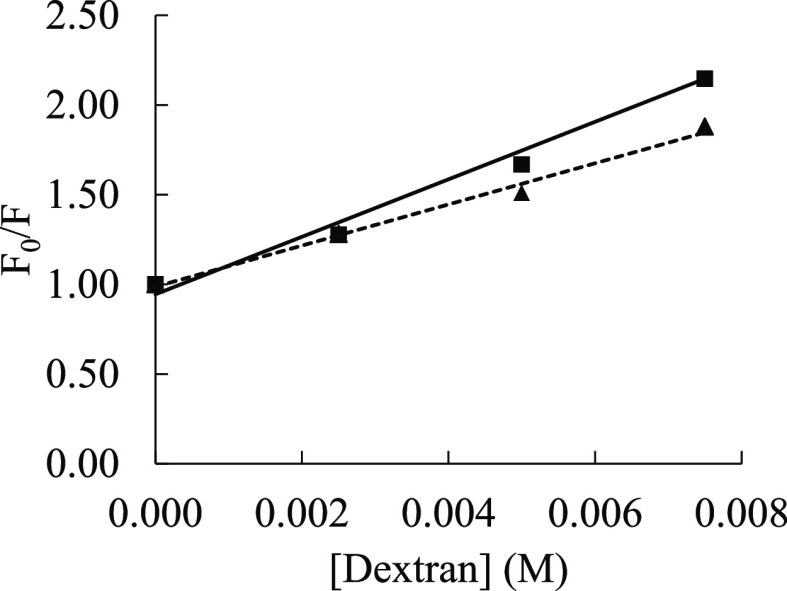
Stern–Volmer plots of tryptophan (10 μM) with varying molar concentrations of dextran 40 at 25 °C (squares, solid line) and 50 °C (triangles, dotted line). The solid and dotted lines represent the lines of best-fitting. The errors were within 0.4 units.

For dextran 40, which also exhibited a linear Stern–Volmer plot, the temperature variation study indicated a reduction in the slope value at 50 °C (115 M^–1^, *R*^2^ = 0.99) relative to that for 25 °C (160 M^–1^, *R*^2^ = 0.98) (Figure 4), suggesting associative interactions and hence static quenching between crowder and fluorophore molecules, and the *K*_SV_ was equated to *K*_S_ (Table 2).

As the *k*_q_ values at 25 and 50 °C for monomer crowders are not significantly different, a quantum chemical study was carried out to further understand the nature of interactions between Trp and crowder molecules. In particular, the associative interactions between Trp and both monomer and polymer crowders were investigated.

### Gibbs’ Binding Free Energy for Crowders with Trp

**Figure 5:**
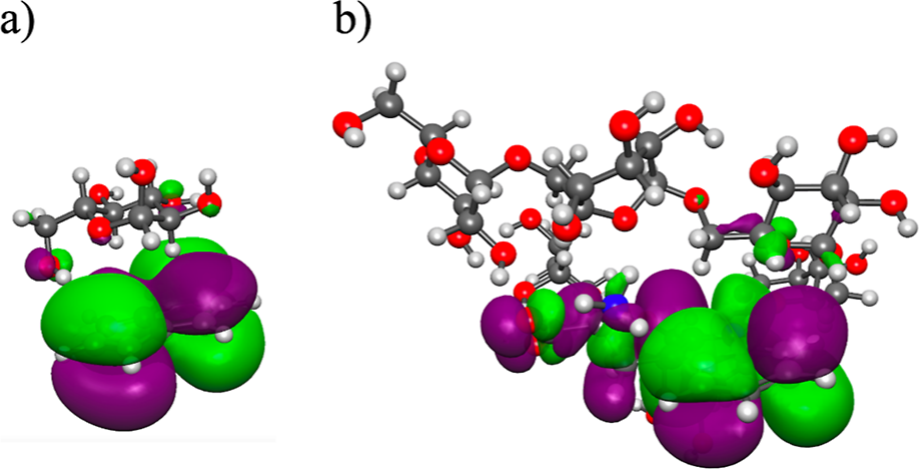
Representative orbital (HOMO) diagrams of crowder complexes: (a) HOMO in the indole:::dextrose complex and (b) HOMO in the Trp:::dextran complex. The CPK coloring method is used to represent carbon (gray spheres), hydrogen (white spheres), nitrogen (blue spheres), and oxygen (red spheres) atoms. The computed models were generated using the M06-2X and 6-31+G(d,p) basis set. The green and purple colors represent the opposite amplitude of the wave function.

**Figure 6:**
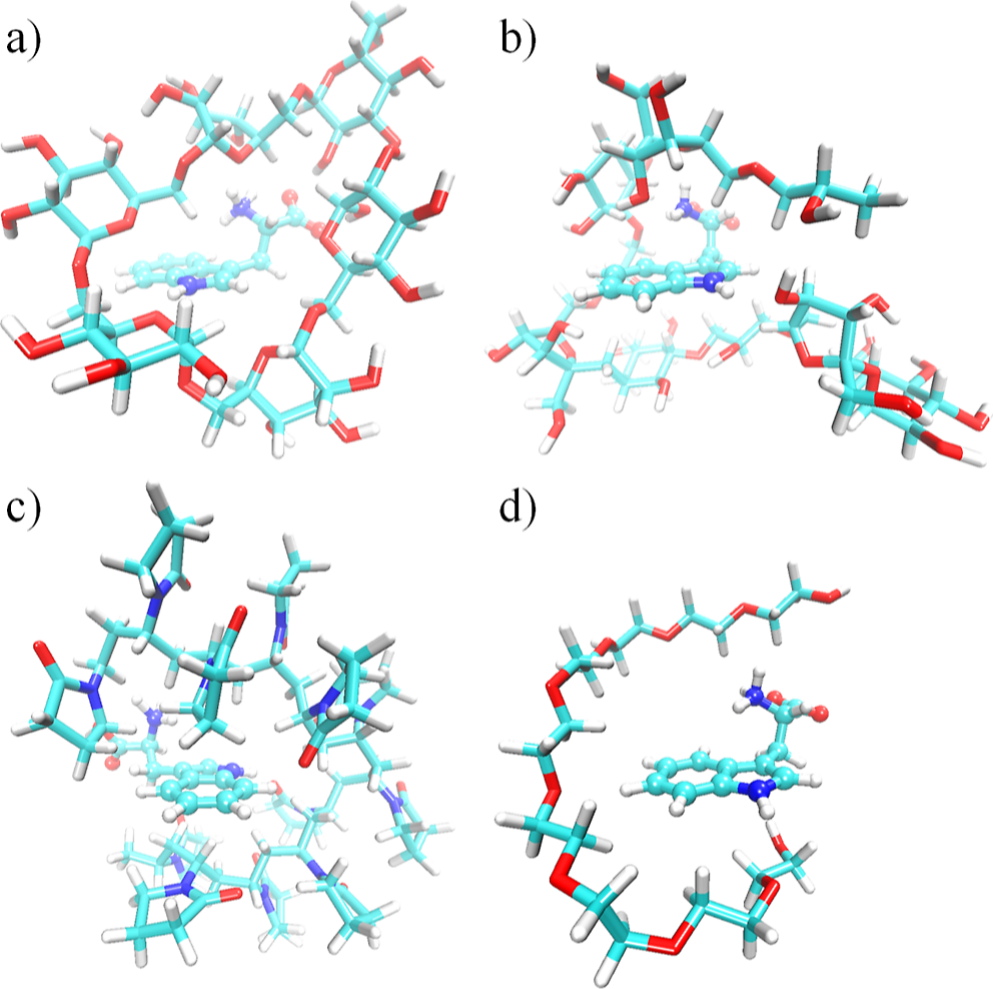
Geometric representations of the optimized Trp-bound polymer crowders: (a) Trp···dextran, (b) Trp···ficoll, (c) Trp···PVP, and (d) Trp···PEG. The Trp is shown as ball and stick, while the polymers are shown as sticks. The carbon, oxygen, nitrogen, and hydrogen atoms are shown in cyan, red, blue, and white colors, respectively.

The quenching mechanism at the molecular level depends on the noncovalent interactions between the crowder and the fluorophore as well as the relative stability of the resultant complex due to solvation. The structure of the Trp-bound synthetic crowders studied using DFT exhibited important differences (Figures 5 and 6). In all calculations, the M06-2X functional has been used because of its reliability to model molecular systems with delocalized electrons.^40^

#### Monomer Crowders

Although σ–π interactions have been reported between a C–H bond and delocalized π-orbitals,^54−59^ the calculated gas phase binding free energies (Δ_bind_*G*° (g)) for indole complexes of the monomer crowders, except with EG, have positive values. This demonstrates the absence of appreciable σ–π interactions; even in the EG complex, a weak association between indole and EG was noted with a Δ_bind_*G*° (g) of −1.0 kcal/mol. As a representative molecular orbital diagram, the HOMO of the indole complexed with dextrose is shown in Figure 5a, which indicates that the frontier orbital is predominantly localized on the indole ring, with little overlap between the conjugated π-orbitals and the C–H σ bonds of the monomer crowder. The computed Δ_bind_*G*° (aq) values for monomer–indole complexes were in the range of 7–11 kcal/mol (Table 4), indicating the absence of complex formation in the aqueous phase.

**Table 4:** Computed Gas- and Aqueous-Phase Gibbs’ Free Energy of Binding of Indole and Monomer Fluorophores Using the M06-2X Functional, SMD implicit solvation, and 6-31+G(d,p) Basis Sets^a^

components of Gibbs’ free energy	dextrose	sucrose	1-ethyl-2-pyrrolidone	ethylene glycol
Δ_bind_*G*° (g)	2.1	1.7	1.5	–1.0
Δ_solv_*G*° (ind)	–6.2	–6.2	–6.2	–6.2
Δ_solv_*G*° (X)	–25.8	–37.0	–7.8	–11.4
Δ_solv_*G*° (ind X)	–23.4	–38.2	–8.7	–9.7
ΔΔ_solv_*G*°	8.6	5.0	5.3	7.9
Δ_bind_*G*° (aq)	10.7	6.7	6.8	6.9

a Following Scheme 1, the Δ_bind_*G*° (g), Δ_bind_*G*° (aq), and ΔΔ_solv_*G*° quantities were calculated using eqs 8–10, respectively.

The decomposition of free energies in Table 4 also illustrates that the overall positive Δ_bind_*G*° (aq) energy is dominated by the large positive (5–9 kcal/mol) solvation Gibbs’ free energy differences (ΔΔ_solv_*G*°). In other words, these results demonstrated that solvated Trp and crowders are stabilized more in the free form than that in their corresponding complex states. The theoretical study therefore demonstrates that solvation plays a dominant role in keeping the fluorophores separated from the crowders. The physical model is fully consistent with the experimentally observed quenching, which originates from the collisional dynamics of Trp and the monomer crowders and solvent molecules.

#### Polymer Crowders

The geometry-optimized structures for the Trp-bound polymers illustrate that dextran (six glucose monomers with 129 atoms), ficoll (three sucrose monomers with 169 atoms), PVP (12 EP monomers with 206 atoms), and PEG (nine EG monomers with 66 atoms) surround the single Trp molecule (Figure 6). In contrast to monomer crowders, dextran exhibited a stronger association with Trp in the aqueous phase (Table 5), while PEG, ficoll, and PVP exhibited no binding. For the Trp:::dextran complex, the gas phase Gibbs binding free energy (Δ_bind_*G*° (g)) was found to be −4.0 kcal/mol, indicating significant association between the Trp and the dextran (Table 5).

**Table 5:** Computed Gas- and Aqueous-Phase Gibbs’ Free Energy of Binding of Tryptophan and Polymer Crowders Using the M06-2X Functional, SMD implicit solvation, and 6-31+G(d,p) Basis Set^a^

components of Gibbs’ free energy	dextran	ficoll	PVP	PEG
Δ_bind_*G*° (g)	–4.0	1.0	–6.3	7.7
Δ_solv_*G*° (Trp)	–16.3	–16.3	–16.3	–16.3
Δ_solv_*G*° (X)	–87.3	–124.3	–69.3	–21.5
Δ_solv_*G*° (ind X)	–101.3	–140.0	–79.6	–36.9
ΔΔ_solv_*G*°	2.3	1.4	6.5	1.1
Δ_bind_*G*° (aq)	–1.7	2.4	0.2	8.8

a A zwitterionic form of the tryptophan was taken for the study. Following Scheme 1, the (Δ_bind_*G*° (*g*), (Δ_bind_*G*° (aq), and ΔΔ_solv_*G*° quantities were calculated using eqs 8–10, respectively.

The optimized structure shows that the crowder wraps up the indole ring from both sides (Figure 6a). Although localized primarily on the indole ring, the HOMO exhibits some delocalization to the adjacent C–C and C–H bonds of the dextran (Figure 5b), indicating the presence of stronger σ–π interactions. The aqueous phase Gibbs binding free energy was computed to be −1.7 kcal/mol (Table 5), which is comparable to the estimated value of −3.0 kcal/mol, obtained using the static quenching constant of 160 M^-1^ (Table 2). The theoretical model therefore indicates that the noncovalent interaction between the indole ring and the dextran plays a predominant role in the complexation. This observation suggests static quenching of Trp by dextran 40.

Interestingly for both Trp:::ficoll and Trp:::PVP complexes (Figure 6b,c), the calculated Δ_bind_*G*° (aq) values were positive but low in magnitude. For the Trp:::ficoll complex, the Δ_bind_*G*° (g) is ∼1 kcal/mol, indicating an insignificant noncovalent interaction between the molecules. The penalty due to solvation (ΔΔ_solv_*G*°) contributes similarly (1.4 kcal/mol), resulting in an overall Δ_bind_*G*° (aq) of 2.4 kcal/mol (Table 5). These results indicate the absence of association between Trp and ficoll in the aqueous phase. For the Trp:::PVP complex, a stronger noncovalent interaction between Trp and PVP is evident from Δ_bind_*G*° (g) of −6.3 kcal/mol. However, the Δ_bind_*G*° (aq) was found to be almost negligible. Comparing the Gibbs free energy components associated with two types of physical processes (Table 5), it appears that the decrease in Gibbs free energy due to complexation of Trp and ficoll (Δ_bind_*G*° (g) = −6.3 kcal/mol) is about the same as the increase in the Gibbs free energy due to the solvation of the complex relative to their separated states (ΔΔ_solv_*G*° = 6.5 kcal/mol). The theoretical model for Trp:::ficoll and Trp:::PVP illustrates the role of solvation effects, which corresponds very well to the static and dynamic components involved in the sphere-of-action mechanism, as observed experimentally.

In contrast, the Trp:::PEG complex (Figure 6d) demonstrated a lack of association between Trp and PEG and an unfavorable solvation component, resulting in a net positive Δ_bind_*G*° (aq) value of 8.8 kcal/mol (Table 5). The large positive Gibbs free energy predicts that in aqueous solution, Trp and the PEG would prefer to remain separated. In the experiment, the Trp:::PEG system exhibits a minimal quenching effect, which can be explained by the quantum chemical model. In summary, the computational study, therefore, provides a physical basis of the difference in the quenching mechanisms between molecular versus macromolecular crowding, and this is similar to the hydration dynamics of interstitial bulk water molecules and those on the crowder/water interface reported earlier.^60^

## Conclusions

The perturbation of fluorescence properties of Trp due to monomer and polymer synthetic crowding agents was investigated, and the physical mechanism of quenching has been characterized. Although not substantial, the quenching of tryptophan fluorescence occurs via mainly dynamic quenching for most of the monomer crowders. On the other hand, polymer crowders were found to involve more complex quenching mechanisms. While PEG 8 did not quench at all, a sphere-of-action mechanism with a significant contribution from dynamic quenching was observed for ficoll 70 and PVP 40. For the polymer crowder dextran 40, the quenching was predominantly static.

The present study offers new physical insights into why polymer crowders are more effective quenchers compared to their respective monomers. Monomer crowders exhibited dynamic quenching (except EG, which resulted in an enhancement in the fluorescence intensity of Trp). Dynamic quenching occurs through collisions with the fluorophore rather than association, which the present study established through the linear Stern–Volmer plot and an increase in quenching at higher temperature. Quantum chemical studies provided the physical basis of the dynamic quenching; the positive Gibbs’ binding free energies demonstrated negligible associative/soft interactions between the tryptophan indole and the monomer crowder molecules.

The fitting of the Stern–Volmer plots exhibited profoundly diverse mechanisms of quenching by polymer crowders. For dextran 40, the physical basis of the fluorescence quenching was determined to be due to an associative interaction, which was confirmed by computed Gibbs binding free energies. In contrast, the mechanistic realm for the Trp quenching in ficoll 70 and PVP 40 appears to be more complex in nature. The quantum chemical computations revealed no associative interactions with Trp, which is consistent with the experimentally observed sphere-of-action mode of quenching by these two polymeric crowders.

In this study, zwitterionic Trp was used as a model, and the observed findings are relevant to the interactions between crowder molecules and the tryptophanyl residue in proteins. This is because previous studies have shown that various ionic forms of Trp exhibit similarity in their spectroscopic behaviors.^61^ The excitation and emission profiles for Trp zwitterion and neutral indole demonstrated that wavelengths of maxima of absorption, excitation, and emission as well as fluorescence efficiencies are extremely close.^61^ However, there is a limitation of the present study in the sense that the zwitterionic Trp cannot completely mimic the interactions between crowders and a tryptophanyl residue in a polypeptide chain because several additional factors, including the fold of the polypeptide, neighboring side chains, solvent accessibility, and the presence of metal ions, can impact the interactions. Thus, further studies with proteins are needed to explore the crowder-induced fluorescence quenching of tryptophanyl residues in polypeptides. Nevertheless, the study demonstrated the diverse fluorescence quenching mechanisms exhibited by synthetic monomers and polymer crowders for free Trp molecules in aqueous solution.

The present study provides molecular-level insights into the preferential exclusion observed in our previous study with bacterial prolyl-tRNA synthetase.^18^ The dynamic fluorescence quenching mechanism exhibited by monomer crowders is consistent with the observed preferential exclusion displayed by monomer crowders; dextrose and sucrose dispersed away from the protein surface, indicating the lack of significant soft interactions between monomer crowders and protein side chains. In contrast, the polymeric crowders would be expected to show some associative trend toward exposed Trp residues. In the broader context, the present study provides new physical insights into the diversity of tryptophan fluorescence quenching due to commonly used synthetic monomer and polymer crowders in water that could have wider implications for designing crowder-based investigations of the structure–function dynamics relationships in enzymes.

## Supporting Information Available

Temperature variation fluorescence study with 10 μM tryptophan in the presence of 300 mg/mL crowders; Tryptophan (10 μM) fluorescence intensity in the presence of varying concentrations of monomer crowders at 25 °C and 50 °C; Stern-Volmer plots of tryptophan (10 μM) with varying concentrations of monomer crowders at 25 °C and 50 °C:(PDF)

## Abbreviations

λ_bcm_: Barycentric mean wavelength
EG: ethylene glycol
EP: 1-ethyl-2-pyrrolidone
PEG: polyethylene glycol
PVP: polyvinylpyrrolidone
ProRS: prolyl-tRNA synthetase
Trp: tryptophan

## References

[ref1] Lakowicz J. R. Principles of Fluorescence Spectroscopy, 2nd ed.; Lakowicz J. R., Ed.; Springer, 2006.

[ref2] Ladokhin A. S. Fluorescence Spectroscopy in Peptide and Protein Analysis. Encyclopedia of Analytical Chemistry; Wiley, 2000.

[ref3] Ladokhin A. S.; Jayasinghe S.; White S. H. How to Measure and Analyze Tryptophan Fluorescence in Membranes Properly, and Why Bother?. Anal. Biochem. 2000, 285 (2), 235–245. 10.1006/abio.2000.4773. 11017708

[ref4] Ghisaidoobe A. B. T.; Chung S. J. Intrinsic Tryptophan Fluorescence in the Detection and Analysis of Proteins: A Focus on Förster Resonance Energy Transfer Techniques. Int. J. Mol. Sci. 2014, 15, 22518–22538. 10.3390/ijms151222518. 25490136 PMC4284722

[ref5] Rossi A. M.; Taylor C. W. Analysis of Protein-Ligand Interactions by Fluorescence Polarization. Nat. Protoc. 2011, 6 (3), 365–387. 10.1038/nprot.2011.305. 21372817 PMC3160472

[ref6] Sridharan R.; Zuber J.; Connelly S. M.; Mathew E.; Dumont M. E. Fluorescent Approaches for Understanding Interactions of Ligands with G Protein Coupled Receptors. Biochim. Biophys. Acta Biomembr. 2014, 1838 (1), 15–33. 10.1016/j.bbamem.2013.09.005. PMC3926105 24055822

[ref7] Ward L. D. [22] Measurement of Ligand Binding to Proteins by Fluorescence Spectroscopy. Methods Enzymol. 1985, 117, 400–414. 10.1016/S0076-6879(85)17024-2. 4079811

[ref8] Sindrewicz P.; Li X.; Yates E. A.; Turnbull J. E.; Lian L. Y.; Yu L. G. Intrinsic Tryptophan Fluorescence Spectroscopy Reliably Determines Galectin-Ligand Interactions. Sci. Rep. 2019, 9 (1), 11851 10.1038/s41598-019-47658-8. 31413267 PMC6694196

[ref9] Alexiev U.; Farrens D. L. Fluorescence Spectroscopy of Rhodopsins: Insights and Approaches. Biochim. Biophys. Acta Bioenerg. 2014, 1837, 694–709. 10.1016/j.bbabio.2013.10.008. PMC3965647 24183695

[ref10] Cohen B. E.; Pralle A.; Yao X. J.; Swaminath G.; Gandhi C. S.; Jan Y. N.; Kobilka B. K.; Isacoff E. Y.; Jan L. Y. A Fluorescent Probe Designed for Studying Protein Conformational Change. Proc. Natl. Acad. Sci. U. S. A. 2005, 102, 965–970. 10.1073/pnas.0409469102. 15657131 PMC545863

[ref11] Jazaj D.; Ghadami S. A.; Bemporad F.; Chiti F. Probing Conformational Changes of Monomeric Transthyretin with Second Derivative Fluorescence. Sci. Rep. 2019, 9, 10988 10.1038/s41598-019-47230-4. 31358790 PMC6662758

[ref12] Zimmerman S. B.; Trach S. O. Estimation of Macromolecule Concentrations and Excluded Volume Effects for the Cytoplasm of Escherichia Coli. J. Mol. Biol. 1991, 222, 599–620. 10.1016/0022-2836(91)90499-V. 1748995

[ref13] Ellis R. J. Macromolecular Crowding: An Important but Neglected Aspect of the Intracellular Environment. Curr. Opin. Struct. Biol. 2001, 11 (1), 114–119. 10.1016/S0959-440X(00)00172-X. 11179900

[ref14] Zhou B. R.; Liang Y.; Du F.; Zhou Z.; Chen J. Mixed Macromolecular Crowding Accelerates the Oxidative Refolding of Reduced, Denatured Lysozyme: Implications for Protein Folding in Intracellular Environments. J. Biol. Chem. 2004, 279 (53), 55109–55116. 10.1074/jbc.M409086200. 15494409

[ref15] Singh P.; Chowdhury P. K. Unravelling the Intricacy of the Crowded Environment through Tryptophan Quenching in Lysozyme. J. Phys. Chem. B 2017, 121, 4687–4699. 10.1021/acs.jpcb.7b01055. 28388056

[ref16] Singh P.; Chowdhury P. K. Crowding-Induced Quenching of Intrinsic Tryptophans of Serum Albumins: A Residue-Level Investigation of Different Conformations. J. Phys. Chem. Lett. 2013, 4 (16), 2610–2617. 10.1021/jz401179z.

[ref17] Gryczynski I.; Wiczk W.; Johnson M. L.; Lakowicz J. R. Lifetime Distributions and Anisotropy Decays of Indole Fluorescence in Cyclohexane/Ethanol Mixtures by Frequency-Domain Fluorometry. Biophys. Chem. 1988, 32, 173–185. 10.1016/0301-4622(88)87005-4. 3251567

[ref18] Adams L. M. M.; Andrews R. J. J.; Hu Q. H. H.; Schmit H. L. L.; Hati S.; Bhattacharyya S. Crowder-Induced Conformational Ensemble Shift in Escherichia Coli Prolyl-TRNA Synthetase. Biophys. J. 2019, 117, 1269–1284. 10.1016/j.bpj.2019.08.033. 31542226 PMC6818166

[ref19] Kuznetsova I. M.; Turoverov K. K.; Uversky V. N. What Macromolecular Crowding Can Do to a Protein. Int. J. Mol. Sci. 2014, 15 (12), 23090–23140. 10.3390/ijms151223090. 25514413 PMC4284756

[ref20] Sanford B.; Cao B.; Johnson J. M.; Zimmerman K.; Strom A. M.; Mueller R. M.; Bhattacharyya S.; Musier-Forsyth K.; Hati S. Role of Coupled Dynamics in the Catalytic Activity of Prokaryotic-like Prolyl-TRNA Synthetases. Biochemistry 2012, 51, 2146–2156. 10.1021/bi300097g. 22356126 PMC3337710

[ref21] Johnson J. M.; Sanford B. L.; Strom A. M.; Tadayon S. N.; Lehman B. P.; Zirbes A. M.; Bhattacharyya S.; Musier-Forsyth K.; Hati S. Multiple Pathways Promote Dynamical Coupling between Catalytic Domains in Escherichia Coli Prolyl-TRNA Synthetase. Biochemistry 2013, 52, 4399–4412. 10.1021/bi400079h. 23731272 PMC3749879

[ref22] Bartholow T. G.; Sanford B. L.; Cao B.; Schmit H. L.; Johnson J. M.; Meitzner J.; Bhattacharyya S.; Musier-Forsyth K.; Hati S. Strictly Conserved Lysine of Prolyl-TRNA Synthetase Editing Domain Facilitates Binding and Positioning of Misacylated TRNA^Pro^. Biochemistry 2014, 53, 1059–1068. 10.1021/bi401279r. 24450765 PMC3986007

[ref23] Strom A. M.; Fehling S. C.; Bhattacharyya S.; Hati S. Probing the Global and Local Dynamics of Aminoacyl-TRNA Synthetases Using All-Atom and Coarse-Grained Simulations. J. Mol. Model. 2014, 20, 2245 10.1007/s00894-014-2245-1. 24810463 PMC4030129

[ref24] Hu Q. H.; Williams M. T.; Shulgina I.; Fossum C. J.; Weeks K. M.; Adams L. M.; Reinhardt C. R.; Musier-Forsyth K.; Hati S.; Bhattacharyya S. Editing Domain Motions Preorganize the Synthetic Active Site of Prolyl-TRNA Synthetase. ACS Catal. 2020, 10, 10229–10242. 10.1021/acscatal.0c02381. 34295570 PMC8293909

[ref25] Ibba M.; Soll D. Aminoacyl-TRNA Synthesis. Annu. Rev. Biochem. 2000, 69, 617–650. 10.1146/annurev.biochem.69.1.617. 10966471

[ref26] Beuning P. J.; Musier-Forsyth K. Species-Specific Differences in Amino Acid Editing by Class II Prolyl-TRNA Synthetase. J. Biol. Chem. 2001, 276 (33), 30779–30785. 10.1074/jbc.M104761200. 11408489

[ref27] Wu J.; Zhao C.; Lin W.; Hu R.; Wang Q.; Chen H.; Li L.; Chen S.; Zheng J. Binding Characteristics between Polyethylene Glycol (PEG) and Proteins in Aqueous Solution. J. Mater. Chem. B 2014, 2, 2983–2992. 10.1039/c4tb00253a. 32261674

[ref28] Jiang Y.; Yan Y. B.; Zhou H. M. Polyvinylpyrrolidone 40 Assists the Refolding of Bovine Carbonic Anhydrase B by Accelerating the Refolding of the First Molten Globule Intermediate. J. Biol. Chem. 2006, 281, 9058–9065. 10.1074/jbc.m507874200. 16459336

[ref29] Ferreira L. A.; Madeira P. P.; Breydo L.; Reichardt C.; Uversky V. N.; Zaslavsky B. Y. Role of Solvent Properties of Aqueous Media in Macromolecular Crowding Effects. J. Biomol. Struct. Dyn. 2016, 34, 92–103. 10.1080/07391102.2015.1011235. 25616385

[ref30] Breydo L.; Sales A. E.; Frege T.; Howell M. C.; Zaslavsky B. Y.; Uversky V. N. Effects of Polymer Hydrophobicity on Protein Structure and Aggregation Kinetics in Crowded Milieu. Biochemistry 2015, 54, 2957–2966. 10.1021/acs.biochem.5b00116. 25919930

[ref31] Kohlmann T.; Goez M. Combined Static and Dynamic Intramicellar Fluorescence Quenching: Effects on Stationary and Time-Resolved Stern-Volmer Experiments. Phys. Chem. Chem. Phys. 2019, 21 (19), 10075–10085. 10.1039/C8CP07486K. 31049527

[ref32] Paterson K. A.; Arlt J.; Jones A. C. Dynamic and Static Quenching of 2-Aminopurine Fluorescence by the Natural DNA Nucleotides in Solution. Methods Appl. Fluoresc. 2020, 8, 025002 10.1088/2050-6120/ab71c3. 32000159

[ref33] Genovese D.; Cingolani M.; Rampazzo E.; Prodi L.; Zaccheroni N. Static Quenching upon Adduct Formation: A Treatment without Shortcuts and Approximations. Chem. Soc. Rev. 2021, 50, 8414–8427. 10.1039/D1CS00422K. 34142693

[ref34] Schlamadinger D. E.; Kats D. I.; Kim J. E. Quenching of Tryptophan Fluorescence in Unfolded Cytochrome c: A Biophysics Experiment for Physical Chemistry Students. J. Chem. Educ. 2010, 87, 961–964. 10.1021/ed900029c. 25593364 PMC4292842

[ref35] Avogadro(Orca). Avogadro: An Open-Source Molecular Builder and Visualization Tool, 2020. Version 1.2.0.

[ref36] Hanwell M. D.; Curtis D. E.; Lonie D. C.; Vandermeersch T.; Zurek E.; Hutchison G. R. Avogadro: An Advanced Semantic Chemical Editor, Visualization, and Analysis Platform. J. Cheminf. 2012, 4, 17 10.1186/1758-2946-4-17. PMC3542060 22889332

[ref37] Frisch M. J.; Trucks G. W.; Schlegel H. B.; Scuseria G. E.; Robb M. A.; Cheeseman J. R.; Scalmani G.; Barone V.; Petersson G. A.; Nakatsuji H.; Gaussian 16, Revision C.01; Gaussian, Inc.: Wallingford, CT, 2016.

[ref38] Kohn W.; Sham L. J. Self-Consistent Equations Including Exchange and Correlation Effects. Phys. Rev. A 1965, 140, 1133–1138. 10.1103/physrev.140.a1133.

[ref39] Zhao Y.; Schultz N. E.; Truhlar D. G. Exchange-Correlation Functional with Broad Accuracy for Metallic and Nonmetallic Compounds, Kinetics, and Noncovalent Interactions. J. Chem. Phys. 2005, 123 (16), 161103 10.1063/1.2126975. 16268672

[ref40] Zhao Y.; Truhlar D. G. The M06 suite of density functionals for main group thermochemistry, thermochemical kinetics, noncovalent interactions, excited states, and transition elements: two new functionals and systematic testing of four M06-class functionals and 12 other functionals. Theor. Chem. Acc. 2008, 120 (1–3), 215–241. 10.1007/s00214-007-0310-x.

[ref41] Hariharan P. C.; Pople J. A. The Influence of Polarization Functions on Molecular Orbital Hydrogenation Energies. Theor. Chim. Acta 1973, 28, 213–222. 10.1007/BF00533485.

[ref42] Marenich A. V.; Cramer C. J.; Truhlar D. G. Universal Solvation Model Based on Solute Electron Density and on a Continuum Model of the Solvent Defined by the Bulk Dielectric Constant and Atomic Surface Tensions. J. Phys. Chem. B 2009, 113 (18), 6378–6396. 10.1021/jp810292n. 19366259

[ref43] Pilati T.; Forni A. SYMMOL: A Program to Find the Maximum Symmetry Group in an Atom Cluster, given a Prefixed Tolerance. J. Appl. Crystallogr. 1998, 31 (3), 503–504. 10.1107/S0021889898002180.

[ref44] Gilbert A. T. B. IQmol Molecular Viewer, 2012.

[ref45] Rappe A. K.; Casewit C. J.; Colwell K. S.; Goddard W. A.; Skiff W. M. UFF, a Full Periodic Table Force Field for Molecular Mechanics and Molecular Dynamics Simulations. J. Am. Chem. Soc. 1992, 114, 10024–10035. 10.1021/ja00051a040.

[ref46] Schultz S. G.; Solomon A. K. Determination of the Effective Hydrodynamic Radii of Small Molecules by Viscometry. J. Gen. Physiol. 1961, 44, 1189–1199. 10.1085/jgp.44.6.1189. 13748878 PMC2195139

[ref47] Armstrong J. K.; Wenby R. B.; Meiselman H. J.; Fisher T. C. The Hydrodynamic Radii of Macromolecules and Their Effect on Red Blood Cell Aggregation. Biophys. J. 2004, 87 (6), 4259–4270. 10.1529/biophysj.104.047746. 15361408 PMC1304934

[ref48] Ling K.; Jiang H.; Zhang Q. A Colorimetric Method for the Molecular Weight Determination of Polyethylene Glycol Using Gold Nanoparticles. Nanoscale Res. Lett. 2013, 8, 538–610. 10.1186/1556-276x-8-538. 24359120 PMC3878192

[ref49] Ying Q.; Chu B. Overlap Concentration of Macromolecules in Solution. Macromolecules 1987, 20, 362–366. 10.1021/ma00168a023.

[ref50] Tang I. H.; Sundari R.; Lintang H. O.; Yuliati L. Polyvinylpyrrolidone as a New Fluorescent Sensor for Nitrate Ion. Malays. J. Anal. Sci. 2016, 20 (2), 288–295. 10.17576/mjas-2016-2002-09.

[ref51] Arık M.; Çelebi N.; Onganer Y. Fluorescence Quenching of Fluorescein with Molecular Oxygen in Solution. J. Photochem. Photobiol., A 2005, 170, 105–111. 10.1016/j.jphotochem.2004.07.004.

[ref52] Gudgin E.; Lopez-Delgado R.; Ware W. R. The Tryptophan Fluorescence Lifetime Puzzle. A Study of Decay Times in Aqueous Solution as a Function of PH and Buffer Composition. Can. J. Chem. 1981, 59, 1037–1044. 10.1139/v81-154.

[ref53] Swaminathan R.; Krishnamoorthy G.; Periasamy N. Similarity of Fluorescence Lifetime Distributions for Single Tryptophan Proteins in the Random Coil State. Biophys. J. 1994, 67, 2013–2023. 10.1016/S0006-3495(94)80685-X. 7858139 PMC1225577

[ref54] Spiwok V. CH/π Interactions in Carbohydrate Recognition. Molecules 2017, 22, 1038–1111. 10.3390/molecules22071038. 28644385 PMC6152320

[ref55] Wimmerova M.; Kozmon S.; Nečasová I.; Mishra S. K.; Komarek J.; Koča J. Stacking Interactions between Carbohydrate and Protein Quantified by Combination of Theoretical and Experimental Methods. PLoS One 2012, 7 (10), e46032 10.1371/journal.pone.0046032. 23056230 PMC3466270

[ref56] Wimmerova M.; Kozmon S.; Nečasová I.; Mishra S. K.; Komarek J.; Koča J. Stacking Interactions between Carbohydrate and Protein Quantified by Combination of Theoretical and Experimental Methods. PLoS One 2012, 7 (10), e46032 10.1371/journal.pone.0046032. 23056230 PMC3466270

[ref57] Karthikeyan S.; Ramanathan V.; Mishra B. K. Influence of the Substituents on the CH.π Interaction: Benzene–Methane Complex. J. Phys. Chem. A 2013, 117 (30), 6687–6694. 10.1021/jp404972f. 23822641

[ref58] Nishio M.; Umezawa Y.; Fantini J.; Weiss M. S.; Chakrabarti P. CH-π Hydrogen Bonds in Biological Macromolecules. Phys. Chem. Chem. Phys. 2014, 16 (25), 12648–12683. 10.1039/C4CP00099D. 24836323

[ref59] Nishio M. The CH/π Hydrogen Bond in Chemistry. Conformation, Supramolecules, Optical Resolution and Interactions Involving Carbohydrates. Phys. Chem. Chem. Phys. 2011, 13 (31), 13873–13900. 10.1039/c1cp20404a. 21611676

[ref60] Verma P. K.; Kundu A.; Cho M. How Molecular Crowding Differs from Macromolecular Crowding: A Femtosecond Mid-Infrared Pump-Probe Study. J. Phys. Chem. Lett. 2018, 9, 6584–6592. 10.1021/acs.jpclett.8b03153. 30380875

[ref61] Bridges J. W.; Williams R. T. The Fluorescence of Indoles and Aniline Derivatives. Biochem. J. 1968, 107, 225–237. 10.1042/bj1070225. 5641878 PMC1198649

