# Supplementary material for: Insights into the Mechanism of Tryptophan Fluorescence Quenching due to Synthetic Crowding Agents: A Combined Experimental and Computational Study
Source: ACS Omega. 2023 Nov 13;8(47):44820–30. doi: 10.1021/acsomega.3c06006 (PMC10688029; doi:10.1021/acsomega.3c06006)

SUPPORTING INFORMATION

Insight into the Mechanism of Tryptophan Fluorescence Quenching due to Synthetic Crowding Agents: A Combined Experimental and Computational Study

Carl J. Fossum, Benjamin O. V. Johnson, Spencer T. Golde, Alexis J. Kielman, Brianna Finke, Macey A. Smith, Harrison R. Lowater, Bethany F. Laatsch, Sudeep Bhattacharyya*, and Sanchita Hati*

Department of Chemistry and Biochemistry, University of Wisconsin-Eau Claire, Wisconsin 54701, U. S. A.

*To whom correspondence should be addressed: S.B.: phone: 715-836-2278; fax: 715-836-4979; email: bhattas@uwec.edu; S.H.: phone: 715-836-3850; fax: 715-836-4979; email: hatis@uwec.edu

Figure S1. Temperature variation study with 10 μM tryptophan in the presence of 300 mg/mL of crowders. The data used here is an average of two trials. The uncertainty in the intensity measurement was within 5 units.

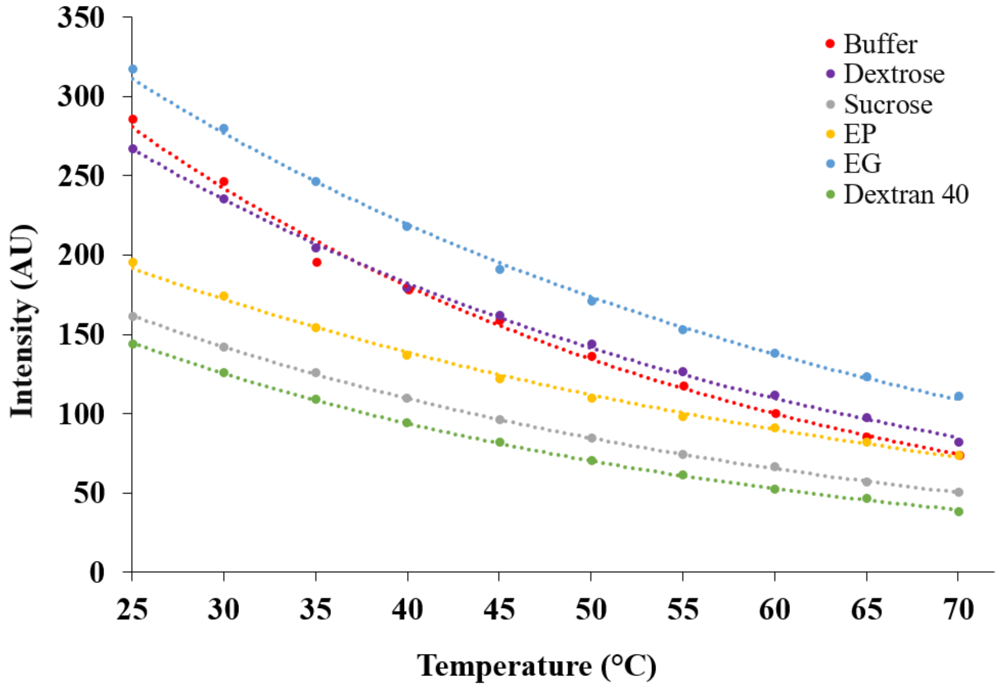

Figure S2. Tryptophan (10 μM) fluorescence intensity in the presence of varying concentrations of monomer crowders at 25 °C (square) and 50 °C (triangle): a) dextrose, b) sucrose, c) EP, and d) EG. The uncertainty in the intensity, measured using two trials, was within 5 units.

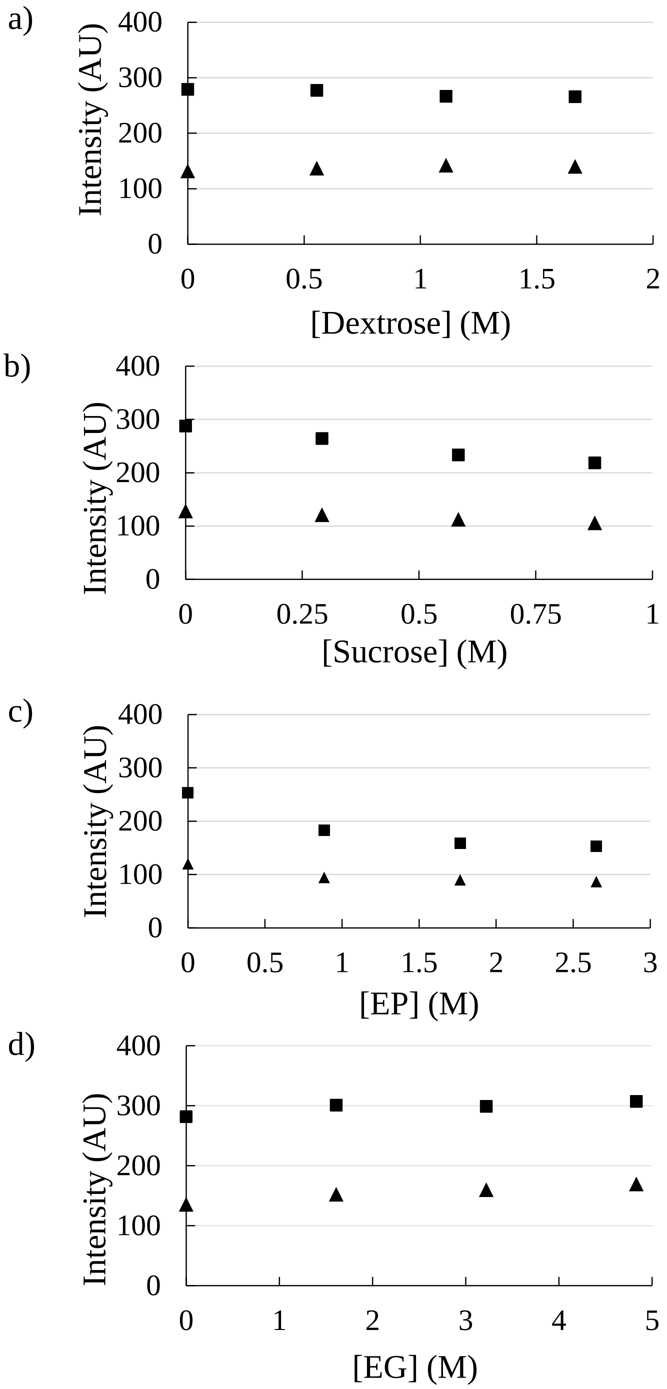

Figure S3. Stern-Volmer plots of tryptophan (10 μM) with varying concentrations of monomer crowders at 25 °C (solid lines) and 50 °C (dotted lines): a) dextrose, b) sucrose, c) EP, and d) EG. Solid and dotted lines represent the line of best-fitting. The data used here is an average of two to three trials. The uncertainty in the intensity measurement was within 0.05 units.

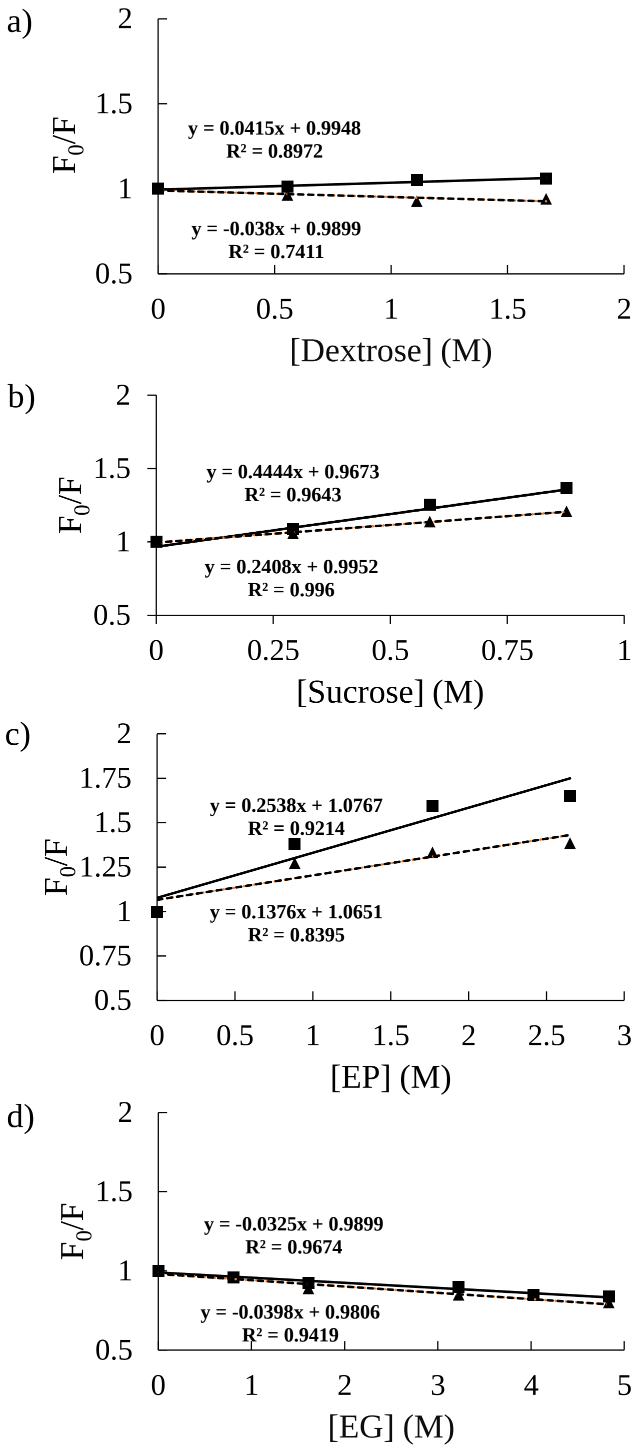

Supplement: Supplementary file 1 — ao3c06006_si_001.pdf [file ao3c06006_si_001.pdf]
